# Effective coping strategies utilised by medical students for mental health disorders during undergraduate medical education-a scoping review

**DOI:** 10.1186/s12909-022-03185-1

**Published:** 2022-02-23

**Authors:** Kamran Sattar, Muhamad Saiful Bahri Yusoff, Wan Nor Arifin, Mohd Azhar Mohd Yasin, Mohd Zarawi Mat Nor

**Affiliations:** 1 Department of Medical Education, School of Medical Sciences, Universiti Sains Malaysia, Kelantan, Malaysia; 2 Biostatistics and Research Methodology Unit, School of Medical Sciences, Universiti Sains Malaysia, Kelantan, Malaysia; 3 Department of Psychiatry, School of Medical Sciences, Universiti Sains Malaysia, Health Campus, Kelantan, Malaysia

**Keywords:** Coping strategies, Medical students, Undergraduate, Medical education, Mental well-being, Mental health disorders, Scoping review

## Abstract

**Background:**

Coping denotes cognitive, emotional and behavioural struggles to tackle a troubled person-environment association. Therefore, coping strategies (CSs) are vital for mental well-being. Widespread research studies have explored this domain, targeting caregivers, nurses, physicians and medical teachers, but limited research has been done to explore the common CSs utilised by medical students at the undergraduate medical education level. Therefore, we aimed to identify the frequently occurring CSs and their effects on mental health disorders (MHDs) through the evidence available in the existing literature.

**Methods:**

For this scoping review, we searched the available literature (articles published from January 1, 1986, to March 31, 2021) on CSs at Google Scholar, PubMed and Scopus using the terms *coping*, *medical students* and *undergraduate medical education*. We included in our search all peer-reviewed journal articles whose central topics were the CSs employed by undergraduate medical students of any age, nationality, race and gender.

**Results:**

From among the 2,134 articles that were found, 24 were ultimately included in the study. The articles were authored in 14 countries, allowing us to gather broader data to answer our research question. The first identified theme (MHDs) had four subthemes: stress (55% of the articles), depression (30%), anxiety (25%) and burnout (15%). The second theme (CSs), on the other hand, had eight subthemes: support seeking (60%), active coping (40%), acceptance (40%), avoidance/denial (40%), substance abuse (35%), faith/religion (25%), sports (25%) and miscellaneous (40%).

**Conclusions:**

Themes and subthemes were identified about the most common CSs utilised by undergraduate medical students to tackle common MHDs in the context of medical education. Among the most used CSs was support (social and emotional) seeking. Teaching medical students how to cope with challenging times is essential.

**Supplementary Information:**

The online version contains supplementary material available at 10.1186/s12909-022-03185-1.

## Background

Medicine is deemed one of the most demanding professional courses [1, 2]. Throughout their education, medical students need help with their educational requirements and coping with their everyday stress. Coping strategies (CSs) for tackling stress and depression are of great importance [3] because the students’ ability to meet the medical-school requirements and to handle challenging situations can affect their academic grades and health [4]. These strategies are behavioural and psychological ways of managing or lessening stressful events [5]. Moreover, S Folkman [6] defines coping as ‘the constant cognitive change and behavioural adaptation when handling specific external and internal demands that are evaluated as something that exceeds the person’s resources. Coping is an active process comprising a sequence of mutual responses through which the person and the situation intermingle and affect each other. It comprises a series of deliberate cognitive and behavioural actions taken to deal with the negative influence of a tense and traumatic situation. Individuals embrace a wide range of cognitive, emotional and behavioural processes while reacting to inner and outer burdens believed to be surpassing their typical assets [6].

Despite the efforts of medical institutes to create a favourable academic culture that would allow medical students to become highly able professionals, high-stress rates still occur in such institutes [7–10], which may have critical consequences on the students’ future professional behaviour [11]. According to Schiller et al. [4], healthcare competence refers to an individual’s modelling of a set of attitudes and values suitable for future healthcare providers. Healthcare authorities thus have the indispensable role of helping ‘students in need’ cope with all the stressors they are bound to encounter in medical school, which may cause them to go on a downhill path academically and in terms of their health. This degradation may jeopardise the students’ mental health during the undergraduate medical years, with lasting and devastating consequences or implications in the years to come (i.e. the clinical years).

Coping in various ways (problem resolving, positive judgement and expression of feelings) tends to be used by students to confront adverse circumstances. Positive reframing, planning and self-distraction are commonly used CSs [12, 13], along with seeking support from peers and seniors, engaging in extracurricular activities, and resorting to religion and humour. These enable students to adapt to adverse circumstances relatively better [14], decrease their anxiety and depression and promote their mental well-being [15].

In recent times, literature discovered numerous MHDs, occurring commonly among healthcare stakeholders. There still lacks a robust exploration of practical CSs utilised by medical students at the undergraduate medical education level. This situation calls for a scoping review with specific objectives identifying practical CSs, utilised globally by undergraduate medical students to target MHDs. This shall have the potential ability to guide those in need. We hope, through this scoping review, once the effective CSs are listed, with evidence from the literature, the students might get help for their mental well-being and shall be able to avoid using harmful CSs which, instead of helping, might augment their problems. Therefore, this scoping review aimed to identify the frequently occurring CSs and their effectiveness on MHDs through the evidence available in the existing literature.

## Methods

We aimed to use a scoping review approach that offers theoretical precision about a particular topic or area of literature by synthesising and analysing a broader array of literature [16]. Thus, we used H Arksey and L O'Malley [17] methodological framework, which has five stages: (1) identifying the research question; (2) identifying relevant studies; (3) selecting studies; (4) charting the data and (5) collating, summarising and reporting the results.

### Stage 1: Identifying the research question

As mentioned earlier, this study aimed to discover the practical CSs used by undergraduate medical students. Our research question was ‘What are the CSs that undergraduate medical students commonly use?’ A functional definition of coping strategy was formulated for the study: patterns used by medical students to control their emotions, feelings and behaviours as they go through different periods of stress.

### Stage 2: Identifying relevant studies

An electronic search for articles published within the period from January 1, 1986, to March 31, 2021, was done on Google Scholar (Google Inc., Mountain View, CA), PubMed (US National Library of Medicine, National Institutes of Health, Bethesda, MD) and Scopus (Elsevier B.V., Amsterdam, Netherlands) databases. After repeated initial searches using various search engines, we found many articles published within the period mentioned above. A three-step search was employed. Firstly, we conducted a primary search on the Google Scholar, PubMed and Scopus databases in April 2021. We evaluated the article titles and abstracts that were found using the index terms employed. Secondly, we searched for full-text articles on all the databases, employing the established keywords and index terms. Thirdly, we investigated the reference lists of all the examined studies to look for further studies. So as not to miss any pertinent research, we applied generally well-defined heading terms in the search, and we conducted the electronic search with the help of a librarian. Finally, we conducted various test searches to refine and improve the search terms. Articles were searched using Medical Subject Headings (MeSH) terms, and PubMed was considered the main source for building a search string that was also to be used for the other databases. Appropriate filters were applied to help retrieve articles based on specific characteristics and specific MeSH terms, publication types or dates to narrow the search (Appendix 2).

**Fig. 1:**
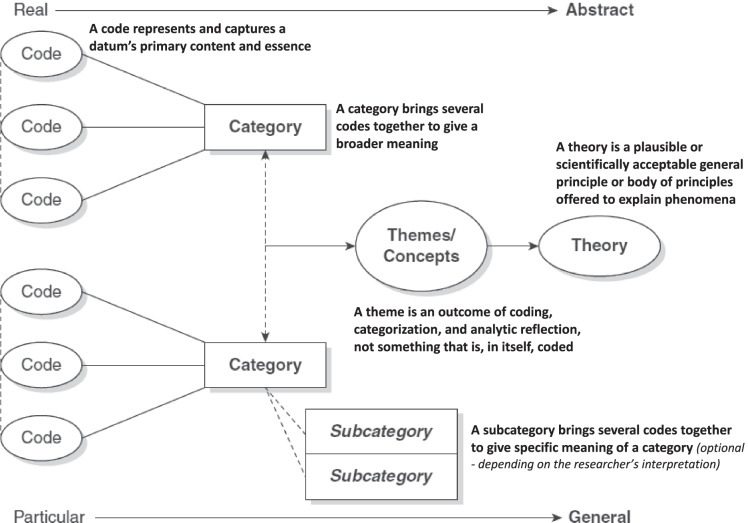
Illustration of a codes-to-theory model for codification and categorisation during the thematic analysis [19]

Additionally, the reference lists of vital articles were also explored for relevant articles that could have been overlooked during the preliminary search. After this secondary search, the articles that met our eligibility criteria were included in the study. The research selection method was reported using the Preferred Reporting Items for Systematic Reviews and Meta-Analysis (PRISMA) flowchart [18], as shown in Fig. 1.

### Stage 3: Selecting studies

**Fig. 2:**
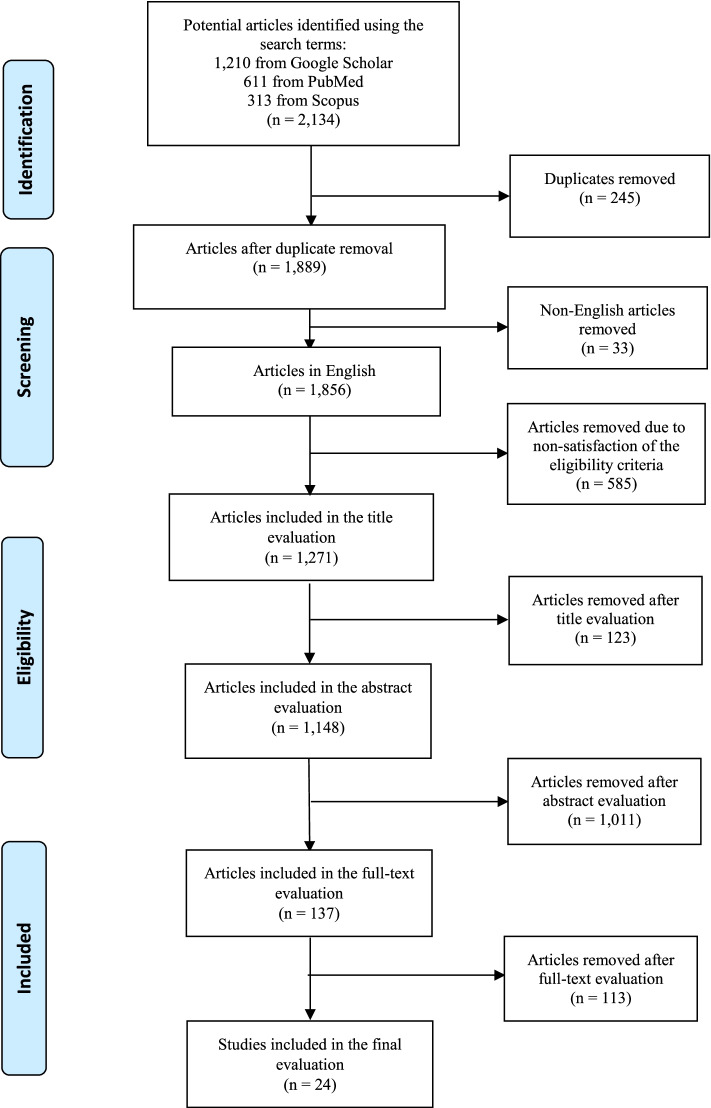
Research consort diagram describing the article selection process employed in this study (PRISMA [21])

The articles that used keywords or words similar to these were recognised at this stage. Three-stage screening (titles, abstract and full text) was carried out. Moreover, the full contents were examined to determine the articles’ fitness for inclusion in our study. The articles were deliberated according to the predetermined eligibility criteria (Table 1). To further ensure the credibility of selected articles, all articles were directly downloaded from the journal site through the full-text link provided by each database, and any retracted/withdrawn articles as well as from the predatory and dubious journals were excluded from the article list. A total of 2,134 articles were initially found based on the search terms used. Then the duplicates were removed, leaving behind 1,889 articles. Of these, 1,148 titles were chosen based on the eligibility requirements, and the abstracts of such articles were recovered. After reviewing the abstracts to determine if they satisfied the suitability measures (Table 1), more articles were removed. Based on the eligibility criteria, 137 abstracts were carefully chosen, and the full texts of the corresponding articles were vetted according to the suitability criteria for full-article selection (Table 1). A total of 24 articles were then included in the study (see Fig. 2). Two reviewers collected full-text articles at this stage [20]. These final search results were exported to the bibliographic software EndNote (Clarivate Analytics, Philadelphia, PA). A third reviewer was available to resolve differences of opinion (but no difference occurred). The inter-rater reliability of the two reviewers was 0.871, with an intraclass correlation coefficient of 0.852 (*p* < 0.001) (Koo & Li, 2016). The study ultimately used 24 articles for data extraction and charting.

**Table 1:** Study eligibility criteria

Steps of determining study suitability	Inclusion criteria
Title suitability	Articles:
	Published within the period from January 1, 1986, to March 31, 2021
	Used the English language
	With the overwhelming theme relating to coping strategies and mental health disorders
Abstract suitability	Abstracts:
	Abstracts of the original article available in a peer-reviewed journal
	Abstracts of articles on studies conducted internationally or nationally
	Abstracts of articles on studies within the context of undergraduate medical education
	Abstracts of articles on studies with medical students and faculty as the participants
	Abstracts of articles on studies that carried out a thorough evaluation of coping strategies and mental health disorders
Full-text suitability	Studies:
	With full-text articles available
	Elaborating effective coping strategies
	With a robust analytical approach of the result(s)
	With a well-designed exploration intervention
	With evidence of evaluation of coping strategies for mental health disorders
	Reporting coping strategies and, or mental health disorders

3 steps of determining study suitability: 1. Title suitability, 2, Abstract suitability, Full-text suitability with details of inclusion criteria applied

### Stage 4: Charting the data

The use of an organised charting method established a priori aided in extracting relevant data from the included studies [17]. Initially, 12 randomly chosen articles were individually studied by two researchers, who drafted the data abstraction form to ensure that their data extraction method is constantly reliable and consistent with the research question. The final form involved two broad headings: study features (authors and year and location of publication) and research area focus (aims, objective, purpose, sample, study design, tools, and CSs used). The coding procedure consisted of three key phases: open coding, creating categories and abstraction [22]. Open coding was carried out by three researchers, who individually read the articles, took down notes and wrote the headings to designate the main research area for each article. The headings and notes were then documented to produce a list of preliminary codes. The list was then refined through a cyclical process by merging similar codes into subcategories. Next, we held debriefing meetings to lay out the necessary explanations, make coding decisions, and develop categories. As we already had a research question, we adopted an inductive approach. Finally, coding was conducted using the inductive qualitative data analysis software NVivo [23]. The details of the extracts from the included articles are exhibited in Appendix 1.

### Stage 5: Collating, summarizing, and reporting the results

At this stage, we recognised the thematic categories in the relevant literature involving various segments, comprising the study methods, evidence, results, and implications. The thematic analysis was conducted in six steps, as suggested by Saldana (2009) [24]: (1) interpreting the texts; (2) tagging the meaningful texts into codes; (3) determining the most significant codes and generating and assembling the associated codes into categories; (4) tagging the categories and determining their relevance and relations; (5) determining the hierarchy, importance or representation of the categories into themes and (6) arranging the results into meaningful themes. Illustration of a codes-to-theory model for codification and categorisation during the thematic analysis is depicted in Fig. 2

When all the data had been assembled, and some opening information had been recognised, we held online meetings to discuss the data analysis and interpretation strategies to employ in the study, the study results obtained, and the writing of this manuscript for publication purposes. The data analysis mainly involved qualitative thematic analysis. To include a statement of the evidence and the possible inconsistencies in the existing knowledge, the interventions and other findings with similar characteristics were arranged thematically. This method helped us gather more structured data and allowed us to come up with the main themes (mental health disorders [MHDs] and CSs) and with substantial results and explicit information connected to our research question (to be explained in the subsequent sections).

## Results

### Literature search

All the initially obtained articles (2,134) were reviewed partly (title and abstract) or in full, and 24 were finally included in our scoping review (Fig. 1). These finally included articles were from 14 countries: five in the United States [4, 19, 25–27]; four in India [28–31]; two each in the United Kingdom [32, 33], Pakistan [34, 35] and Germany [36, 37] and one each in Hong Kong [38], Nepal [13], Austria [3], Iran [39], Romania [40], Malaysia [41], Turkey [42], Iraq [43] and Saudi Arabia [44].

### Study characteristics of the included articles

The included studies were cross-sectional [3, 13, 19, 27, 29, 30, 34, 35, 37, 39, 41, 43, 44], longitudinal [32], longitudinal prospective [31], retrospective [25], qualitative [33] and mixed-method [42] studies, with sample sizes ranging from 122 to 622 medical students. The instruments that were used by the included studies for various variables are listed in Table 2.

**Table 2:** Instruments used by the included studies for various variables

Variables explored	Inventory/survey	Studies
**Mental health disorders**^**a**^	Beck Depression Inventory	[38]
	General Health Questionnaire and list of potential stressors	[32]
	Source and Severity of Stress Scale	[29]
	Questionnaire for perceived stress scale, Hamilton Anxiety Rating Scale, sources of stress	[31]
	Stress and coping questionnaire, depression screening	[3]
	Maslach Burnout Inventory–Student Version	[36]
	Major Depression Inventory, Beck Anxiety Inventory	[37]
	Malay version of Hospital Anxiety Depression Scale	[41]
	Cognitive Emotion Regulation Questionnaire	[40]
	Student Adaptation to College Questionnaire	[42]
	Maslach Burnout Inventory–Human Services Survey	[19]
	Medical Student Stress Questionnaire	[43]
	Kessler 10–Psychological Distress Questionnaire	[44]
**Coping strategies**^**b**^	Coping inventory	[13, 19, 29, 31, 32, 42, 44]
	Coping behaviour inventory	[43]
	Coping strategy scales	[38]
	Ways-of-coping scale	[4]
	Standard questionnaire on religious coping methods	[39]
	Potential functional- and dysfunctional-behaviour-based coping strategies questionnaire	[36]
	Problem-focused styles-of-coping inventory	[37]
	Malay version of Brief Religious Coping Scale	[41]
	A strategic approach to coping scale	[40]
	Self-developed questionnaire	[44]

^a^Stress, depression, anxiety, burnout

^b^Support seeking; active coping; acceptance; avoidance/denial; substance abuse; faith/religion; sports, leisure, games (mobile device/personal computer) and miscellaneous

### Results of the thematic analysis

**Fig. 3:**
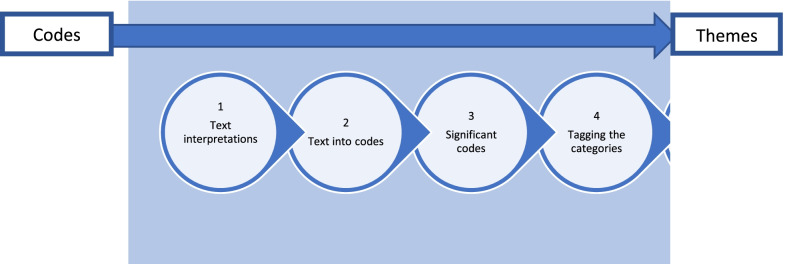
Steps of the ‘codes to theory’ process. Saldana [24] 6 step process carried out for thematic analysis

With a thematic analysis, we looked for patterns in the data that can be used to shed light on our research. Analysis was conducted in different stages. This process of ‘code-to-theory’ is illustrated in Fig. 3, and the steps carried out were: (1) interpreting the texts; at this stage, authors read and re-read the texts from the available studies. Making notes and jotting down early impressions were found to be beneficial. (2) tagging the meaningful texts into codes; at this point, we began to organize our data in a meaningful and systematic manner. Every line of text was not coded. We were interested in answering specific research questions, so we used a theoretical thematic analysis rather than inductive. As a result, we coded any data segment relevant to our research question or captured something intriguing about it. This step reduced a significant volume of data into manageable parts of information relevant to the needed perspective and research questions. (3) determining the most significant codes and generating and assembling the associated codes into categories; here, we were able to find the most frequent codes without losing meaningful data. This also helped us capture significant concerns, further developing into prominent categories. (4) tagging the categories and determining their relevance and relations; at this point, the authors work towards finding out frequent categories and the underlying meaning. It was also established which categories were related to each other and were oriented to a particular concept. (5) determining the hierarchy, importance, or representation into themes. We had the most significant themes at this crucial stage with an order of occurrence, representing the extracted information from the included studies. The significance was based on their representation concerning the research questions.

(6) arranging the results into meaningful themes. At this stage, we found a fitting answer to our research question in the form of extracted points relating to the two main themes: MHDs and CSs. These two themes embody the most discussed issues about psychological morbidity with coping in the undergraduate medical-education context within the published literature. The identified themes yielded subthemes. Theme 1 (MHDs) had four subthemes: burnout, stress, depression and anxiety. Theme 2 (CSs), on the other hand, had eight subthemes: support (social and emotional) seeking; active coping; acceptance; avoidance/denial; substance abuse; faith/religion; sports, leisure, games (mobile device/personal computer [PC]) and miscellaneous.

### Frequently addressed mental health disorders

Mental health issues are becoming more common among medical students and can have a detrimental effect on their overall mental health, affecting their everyday learning and practice tasks. As shown in Table 3, stress, depression, anxiety, and burnout emerged as MHD (theme 1) subthemes in this study. MHDs and their subthemes, when faced by undergraduate medical students, were tackled with various CSs (to be described in the subsequent section).

**Table 3:** Mental health disorders (frequency) targeted in the included studies

Mental health disorders	Percentage of the 24 included studies	Studies
Stress	55%	[3, 13, 25, 28, 29, 31, 32, 34, 35, 43, 44]
Depression	30%	[3, 27, 34, 37, 38, 41]
Anxiety	25%	[31, 34, 37, 40, 41]
Burnout	15%	[19, 27, 36]

Mental health disorders (stress, depression, anxiety and burnout) their percentage within the included studies

### Frequently used coping strategies

All the 24 included studies highlighted the various CSs employed by undergraduate medical students to tackle challenging events. We identified the CSs employed as a theme in the included studies and classified these into eight subthemes: support (social and emotional) seeking; active coping; acceptance; avoidance/denial; substance abuse; faith/religion; sports, leisure, games (mobile device/PC) and miscellaneous. Table 4 shows the CDs frequently reported by undergraduate medical students.

**Table 4:** Frequently reported coping strategies in the included studies

Coping strategies	Percentage of the 24 included studies	Studies
Support (social and emotional) seeking	60%	[3, 4, 13, 28–30, 33–36, 39, 44]
Active coping	40%	[3, 4, 13, 26, 30, 31, 34, 42]
Acceptance	40%	[4, 13, 26, 30, 34, 40, 42, 44]
Avoidance/denial	40%	[4, 27, 30, 31, 34, 38, 43, 44]
Substance abuse	35%	[3, 28–30, 34, 36, 42]
Faith/religion	25%	[3, 30, 34, 39, 41]
Sports, leisure, games (mobile device/personal computer)	25%	[3, 28, 35, 36, 44]
Miscellaneous^a^	40%	[4, 13, 28–30, 34, 35, 42]

^a^Miscellaneous includes sleeping, resorting to humour, engaging in self-blame, isolating/distancing oneself from others, venting and distracting oneself

### Effective coping strategies

CSs have effects (positive or negative) on individuals’ mental health. We found that some of them can successfully decrease the adverse effects of stresses on well-being, whereas others cannot. Table 5 summarises the effective CSs drawn from the included studies.

**Table 5:** Most effective coping strategies with geographic locations

Positive coping strategy^a^	Negative coping strategy^b^	Study	Country (region)
Planned problem solving	Escape, avoidance	[25]	USA (North America)
Problem-focused activities	Brooding over problems	[38]	Hong Kong (Eastern Asia)
Active coping strategies (acceptance, planning, self-distraction)	Avoidance (denial, alcohol use, drug use, behavioural disengagement)	[32]	UK (Northern Europe)
Spending time with friends	Isolation	[35]	Pakistan (South-Central Asia)
Positive reframing	Taking drugs, alcohol	[13]	Nepal (South-Central Asia)
Talking to friends	Engaging in physical exercise, consuming tobacco	[28]	India (South-Central Asia)
Communicating with others	Detached coping	[26]	USA (North America)
Positive coping, religious coping	Substance use, negative coping, blaming	[29]	India (South-Central Asia)
Adaptive coping	Maladaptive coping	[30]	India (South-Central Asia)
Approach-oriented coping	Being avoidance-oriented	[27]	USA (North America)
Positive religious coping	Negative religious coping	[39]	Iran (South-Central Asia)
Seeking support (from friends, family and fellow students), engaging in relaxing exercises, engaging in sports	Taking tranquillisers, stimulants, alcohol	[36]	Germany (Western Europe)
Planned problem solving, seeking social support and engaging in positive reappraisal (e.g. by decreasing overtime)	Emotional escape-avoidance and distancing (e.g. by increasing overtime)	[4]	USA (North America)
Reflective coping	Suppression and reaction	[37]	Germany (Western Europe)
Active coping	Substance use	[31]	India (South-Central Asia)
Positive religious coping	Negative religious coping	[41]	Malaysia (Southeast Asia)
Positive reappraisal and refocusing on planning and action	Avoidance, rumination and catastrophising	[40]	Romania (Eastern Europe)
Religion	Substance abuse	[34]	Pakistan (South-Central Asia)
*Internal strategies* (reinterpretation, normalisation, staying busy and enduring negative emotions)	–	[33]	UK (Northern Europe)
*External strategies* (speaking to someone)			
Learning to live in the current COVID-19 situation and accepting it	Refusing to acknowledge the current COVID-19 situation	[44]	Saudi Arabia (Arabian Peninsula, Middle East)
Problem-solving behaviour	Avoidance behaviours	[43]	Iraq (Middle East, Western Asia)
Adaptive coping/positive reinterpretation/planning	Maladaptive coping (behavioural disengagement and denial)	[19]	USA (North America)
Positive thinking and active coping	Substance use	[3]	Austria (Western Europe)
Planning	Substance use	[42]	Turkey (Southeastern Europe, Western Asia)

^a^Positive (most frequent/highest scoring/supportive/functional)

^b^Negative (less frequent/lowest scoring/non-supportive/dysfunctional)

## Discussion

To the best of our knowledge, this study was the first to explore the CSs most frequently used by undergraduate medical students for MHDs, and the most effective ones among them. The central imperative themes extracted from the 24 included studies were primarily on how essential CSs are for undergraduate medical students in times of need. Medical students face various challenges that affect their mental health during their academic life, and CSs are basic methods of coping with MHDs [45].

Most of the studies that were included in our study highlighted various MHDs (stress [3, 13, 25, 28, 29, 31, 32, 34, 35, 43, 44], depression [3, 27, 34, 37, 38, 41], anxiety [31, 34, 37, 40, 41] and burnout [19, 27, 36]). It was noted that the subtheme of stress was frequently addressed and is common in professions requiring continuous near-human contact and emotional commitment. Various types of stress are frequently reported in the literature, including those from peers, daily life and the environment [46] and those involving finding new friends and learning new responsibilities [47]; finding oneself in unfamiliar circumstances, having to relate with people one does not know and economic worries [48]; interactions with friends and tutors [49] and poor relationships with the clinical staff [50]. It is important to address the stress experienced by medical students. It can undeniably influence their current academic performance and may lead to substance abuse [51], resulting in their declined empathy in their clinical years [11]. Stress has damaging effects on cognitive performance (e.g. attention and decision making) and mental and physical well-being when it is excessive or unresolved due to poor coping [52]. Studies have found that stress does not stand alone, and high-stress levels can lead to other forms of MHD, such as depression, anxiety and burnout [53].

Depression is associated with stress [3, 54]. As medical students are exposed to death and sadness, they are more likely to experience depression than other students [55] and the general population [56], which affects their quality of life [57]. Anxiety is also coupled with isolation and may harm students’ self-confidence and affect their academic performance [31]. Some of the included studies also addressed burnout as a form of MHD. Physician wellness has earned much attention in the past few years [19]. The World Health Organization recently documented burnout as an in-service medical syndrome [58]. It is defined as a state of physical fatigue and mental distress caused mainly by work and professional demands [59]. However, medical students also experience burnout and are often hesitant to seek counselling and treatment. Hence, burnout tends to damage their health. Medical students suffer from this stigma, which often stops them from availing themselves of mental health services [27].

The most significant finding in this study is that undergraduate medical students go through challenging times in medical school. The included studies found that availing of different types of support (social and emotional*,* from friends, family members and fellow students) is the CS most often used by medical students [3, 4]. The pattern of actions carried out when one seeks social help is referred to as support utilisation [60]. Many medical students in the included studies joined study groups for social assistance to study. However, seeking social support involves challenges, such as the long duration of academic study and the tendency for medical students to lose their connections with their peers who had given them social support in medical school.

We discovered, however, that medical students use various CSs other than seeking support, such as active coping, acceptance, avoidance, substance abuse, religious coping and engaging in sports. The students in the included studies differed broadly in their CS use. The students who used active coping showed higher academic performance than those who used other CSs [4]. The avoidance-oriented CSs were deemed less helpful than the engagement-oriented CSs and, interestingly, were used by the lower-performing students [61]. A balanced attitude (between the approach and avoidance strategies) was shown to attract most students and was effective. In contrast, when used alone, maladaptive coping (avoidance/denial) proved to be less effective in tackling burnout and depression [27].

The literature reports substance abuse among medical students. Medical students’ use of illegal drugs is comparable to that of their age-related peers. Statistics show that the medical students using these drugs have been using them even before they enrolled in medical school [62, 63]. Substance abuse is used to relieve distress and cope with isolation, stress, anxiety and depression and is associated with psychosocial impairments such as study stress and job pressure [64]. N Demiral Yilmaz, H Sahin and A Nazli [42] found that substance abuse is among the CSs used by international students. They stated that the maximum mean score in Brief COPE fits in with the Planning subscale and that the bottom is linked to the Substance Use subscale [42].

Religion and mental health have long been seen as partners, with constructive faith coping offering much-needed comfort during difficult times [65, 66]. Pargament explains that religious coping means using faith as a source of strength during stressful times [67]. However, the studies on the effectiveness of religious coping for people in traumatic circumstances have shown mixed results. According to Francis et al. (2019), religious coping is effective in the Southeast Asian region [41] because it is the conventional geographical centre point for many religions and cultures.

Positive religious coping entails evaluating challenges considering God’s providence and establishing a stable relationship with God. On the other hand, negative religious coping is maladaptive and sees obstacles as a form of retribution for one’s disobedience of God’s commands [68]. The medical students in the included studies showed more positive than negative religious coping, as demonstrated by their relevant mean score [41].

Engaging in sports and leisure activities is also among the useful CSs utilised by medical students. Engaging in sports (or exercising) was placed third among the CSs used by such students and seen as a helpful CS [28]. In another study, the most repeatedly mentioned CS was spending time with one’s friends, followed by sleeping, listening to music, engaging in sports and isolating oneself. The female participants opted to study and sleep, whereas the male participants preferred to socialise with their friends, engage in sports or detach themselves from others [35].

We found that social support negatively correlates with depression and exhaustion in medical students. A considerable body of research has emerged showing the positive impact of social support on well-being in healthy and ill people [69–71]. In exploring the relationships between depression, anxiety, family functioning, social support and coping styles among Chinese medical students, it was found that depression is significantly negatively correlated with social support [72].

We also found that the medical students in the included studies explicitly chose active coping, acceptance and avoidance while tackling various MHDs. A study [73] surveyed undergraduate and graduate entry students. Both groups registered similar levels of MHDs, but the strategies that they used to cope with such MHDs differed. The graduate entry students were more likely to use active coping and positive reframing but were more likely to use substances (alcohol and drugs) to help them cope. In contrast, the undergraduate students were more likely to use religion (i.e. praying or meditating) as a way of coping. Other studies have also shown that undergraduate medical students in the United Kingdom use much alcohol, cannabis and other illicit substances [74, 75]. The occurrence of mental illness and drug abuse among medical students can vary from that in the general population in some ways. For example, American medical students use alcohol, benzodiazepines and prescription opiates at higher rates than other similar-age cohorts [76]. A predicted reason that substance abuse is common among medical students is the inappropriate teaching about substance abuse. Medical students generally accept that the teaching of drug abuse in medical schools is generally insufficient [77]. Overall, however, drug abuse is linked to psychosocial impairments such as study stress and job pressure, and illegal drugs are taken to relieve discomfort and cope with isolation, stress, anxiety and depression [64].

Engaging in physical activities, sports, and socialising is essential for personal development and growth [78]. Engaging in sports has been shown to have stress relief and overall health benefits. We likewise found that participating in sports is also used as a CS by medical students. It was reported that participating in sports is a common CS among them [35]. A study from Saudi Arabia reported sports as among the leisure activities engaged in by undergraduate medical students as CSs [79].

CSs have effects (positive or negative) on individuals’ mental health. We found that they work both ways: some CSs can successfully lower the adverse effects of stress on mental well-being, whereas others cannot. The extent to which CSs can detach people from worries may be associated with their perception of their control over the events causing them to worry and maybe favourably connected with their psychological well-being.

Being able to cope with stress positively can help medical students prevent the occurrence of dire implications for their mental health. This can help them feel better physically and psychologically and positively affect their performance at their best. Research shows that students with an active coping style (those who can confront problems positively and straightforwardly) have less emotional distress [80].

Sometimes, one may find it hard to resist engaging in a negative CS that will give one instant relief but may pose bigger challenges for oneself down the road. The well-known inventory for coping (Brief COPE) developed by Pargament [67, 68] allows the assessment of negative and positive coping, with equal distributions of items (i.e. seven for each). This inventory appears to be frequently utilised by studies (see Appendix 1). The negative CSs (shown and expressed in Table 5 as less frequent/lowest scoring/non-supportive/dysfunctional) reported by the included studies were ‘escape and avoidance’ [4, 19, 25–27, 32, 40, 43], ‘drugs and alcohol’ [3, 13, 29, 31, 32, 34, 36, 81] and ‘negative religious couping’ [39, 41].

The included studies explained that perceived stress is linked to academic, psychosocial and environmental stressors. Therefore, the academic curriculum and assessment patterns must be reframed, and counselling cells must be established within the school. In addition, leisure and sports activities must be incorporated into the medical curriculum to make medical education less tense, which can be achieved by providing additional time and resources for leisure and sports.

### Future directions

Despite the substantial body of research demonstrating a positive relationship between support and happiness, we still know very little about the mechanisms by which support influences psychological and physical health. More attention should be given to those who are disabled by drug abuse or mental illness, and renewed efforts are needed to emphasise their prevention and early diagnosis and the care given to those afflicted with them. According to our findings, health support programs and proper guidance on using effective and positive CSs will improve undergraduate medical students’ psychological resilience. That is, their mental health and psychological well-being will benefit from these. However, the authors believe that a meta-analysis is needed to shed more light on this topic.

### Strengths and limitations

This study used a well-known and commonly accepted approach for scoping several articles to synthesise the available data on the issue of the CSs most frequently used by undergraduate medical students. We had two reviewers at every level, and searching for original studies from more than one database gives evidence of our study’s meticulousness, which can be considered a strength of our study. Nevertheless, we cite herein the limitations of our study. Firstly, only English-language research articles were reviewed. Because coping has grown as a universally popular domain for researchers, there may be relevant studies in languages other than English.

Secondly, this scoping review utilised three central databases accessible to the authors when this review was conducted; therefore, including more databases might enrich the review outcomes. Finally, despite the extensive search approach and inclusion criteria, we did not find many studies from Middle Eastern and Southeast Asian countries. This may also be linked to English being not the first language in such countries.

## Conclusion

Undergraduate medical students worldwide experience a wide range of MHDs (stress, depression, anxiety, and burnout) and during such challenging times, try a variety of CSs. The most common is support (social and emotional) seeking, active coping, acceptance, avoidance, substance abuse, religious coping and sports engagement. It is important to emphasise that aside from educating medical students in a professional medical course, their quality of life during their medical training must also be considered. Thus, teaching medical students how to deal with adversity is critical. Individual and administrative involvement is essential for preventing MHDs among medical students.

## Supplementary Information

**Additional file 1. **

**Additional file 2. **

## Data Availability

All data generated or analysed during this study are included in this published article and its supplementary information files. The corresponding author can be contacted if any further clarification is required.

## Abbreviations

CSs: Coping Strategies
PRISMA: Preferred Reporting Items for Systematic Reviews and Meta-Analysis
Misc: Miscellaneous

## References

[1] Waghachavare VB, Dhumale GB, Kadam YR, Gore AD (2013). A Study of Stress among Students of Professional Colleges from an Urban area in India. Sultan Qaboos Univ Med J.

[2] Behere SP, Yadav R, Behere PB (2011). A comparative study of stress among students of medicine, engineering, and nursing. Indian J Psychol Med.

[3] Steiner-Hofbauer V, Holzinger A (2020). How to Cope with the Challenges of Medical Education? Stress, Depression, and Coping in Undergraduate Medical Students. Acad Psychiatry.

[4] Schiller JH, Stansfield RB, Belmonte DC, Purkiss JA, Reddy RM, House JB, Santen SA (2018). Medical Students' Use of Different Coping Strategies and Relationship With Academic Performance in Preclinical and Clinical Years. Teach Learn Med.

[5] Di Mattei VE, Prunas A, Novella L, Marcone A, Cappa SF, Sarno L (2008). The burden of distress in caregivers of elderly demented patients and its relationship with coping strategies. Neurol Sci.

[6] Folkman S: Stress: Appraisal and Coping. In: Encyclopedia of Behavioral Medicine. edn. Edited by Gellman MD, Turner JR. New York, NY: Springer New York; 2013: 1913–1915.

[7] Guthrie EA, Black D, Shaw CM, Hamilton J, Creed FH, Tomenson B (1995). Embarking upon a medical career: psychological morbidity in first year medical students. Med Educ.

[8] Sarkar S, Gupta R, Menon V (2017). A systematic review of depression, anxiety, and stress among medical students in India. J Ment Health Hum Behav.

[9] Hope V, Henderson M (2014). Medical student depression, anxiety and distress outside North America: a systematic review. Med Educ.

[10] Dyrbye LN, Thomas MR, Shanafelt TD (2006). Systematic review of depression, anxiety, and other indicators of psychological distress among U.S. and Canadian medical students. Acad Med.

[11] Woloschuk W, Harasym PH, Temple W (2004). Attitude change during medical school: a cohort study. Med Educ.

[12] Rashid F, Shahid Z, Atif I, Wazir S, Khalid M, Hamid F (2019). Coping strategies of depression, anxiety and stress amongst medical students from different colleges of Rawalpindi and Islamabad. Pakistan Rawal Medical Journal.

[13] Sreeramareddy CT, Shankar PR, Binu VS, Mukhopadhyay C, Ray B, Menezes RG (2007). Psychological morbidity, sources of stress and coping strategies among undergraduate medical students of Nepal. BMC Med Educ.

[14] Curlin FA, Lantos JD, Roach CJ, Sellergren SA, Chin MH (2005). Religious characteristics of U.S. physicians: a national survey. J Gen Intern Med.

[15] Maslach C, Schaufeli WB, Leiter MP (2001). Job Burnout. Annu Rev Psychol.

[16] Khalil H, Peters M, Godfrey CM, McInerney P, Soares CB, Parker D (2016). An Evidence-Based Approach to Scoping Reviews. Worldviews on Evidence-Based Nursing.

[17] Arksey H, O'Malley L (2005). Scoping studies: Towards a methodological framework. Int J Soc Res Methodo.

[18] Moher D, Liberati A, Tetzlaff J (2009). Altman DG Preferred reporting items for systematic reviews and meta-analyses the Prisma statement. PLoS Med.

[19] Shoua-Desmarais N, von Harscher H, Rivera M, Felix T, Havas N, Rodriguez P, Castro G, Zwingli E (2020). First year burnout and coping in one US medical school. Acad Psychiatry.

[20] Levac D, Colquhoun H, O'Brien KK (2010). Scoping studies: Advancing the methodology. Implement Sci.

[21] Saldana J: The Coding Manual for Qualitative Researchers, 2nd edn: Sage Publications; 2013.

[22] Elo S, Kyngas H (2008). The qualitative content analysis process. J Adv Nurs.

[23] Wong L (2008). Data analysis in qualitative research: a brief guide to using nvivo. Malays Fam Physician.

[24] Saldana J (2009). The Coding Manual for Qualitative Researchers.

[25] Wolf TM, Faucett JM, Randall HM, Balson PM (1988). Graduating medical students' ratings of stresses, pleasures, and coping strategies. J Med Educ.

[26] Shapiro J, Lie D (2009). A comparison of medical students' written expressions of emotion and coping and standardized patients' ratings of student professionalism and communication skills.

[27] Thompson G, McBride RB, Hosford CC, Halaas G (2016). Resilience Among Medical Students: The Role of Coping Style and Social Support. Teach Learn Med.

[28] Shah C, Trivedi RS, Diwan J, Dixit R, Anand AK (2009). Common stressors and coping of stress by medical students. J Clin Diagn Res.

[29] Cherkil S, Gardens SJ, Soman DK (2013). Coping styles and its association with sources of stress in undergraduate medical students. Indian J Psychol Med.

[30] Sen S, Pal D, Hazra S, Pandey GK (2015). Spiritual health of students in government medical colleges of Kolkata and their coping skills in a crisis situation. Indian J Public Health.

[31] Balaji NK, Murthy PS, Kumar DN, Chaudhury S (2019). Perceived stress, anxiety, and coping states in medical and engineering students during examinations. Ind Psychiatry J.

[32] Moffat KJ, McConnachie A, Ross S, Morrison JM (2004). First year medical student stress and coping in a problem-based learning medical curriculum. Med Educ.

[33] Trivate T, Dennis AA, Sholl S, Wilkinson T (2019). Learning and coping through reflection: exploring patient death experiences of medical students. BMC Med Educ.

[34] Rashid F, Shahid Z, Atif I, Wazir S, Khalid M, Hamid F (2019). Coping strategies of depression, anxiety and stress amongstmedical students from different colleges of Rawalpindi and Islamabad. Pakistan Rawal Medical Journal.

[35] Shaikh BT, Kahloon A, Kazmi M, Khalid H, Nawaz K, Khan N, Khan S (2004). Students, stress and coping strategies: a case of Pakistani medical school. Educ Health (Abingdon).

[36] Erschens R, Loda T, Herrmann-Werner A, Keifenheim KE, Stuber F, Nikendei C, Zipfel S, Junne F (2018). Behaviour-based functional and dysfunctional strategies of medical students to cope with burnout. Med Educ Online.

[37] Akhtar M, Herwig BK, Faize FA (2019). Depression and Anxiety among International Medical Students in Germany: The Predictive Role of Coping Styles. J Pak Med Assoc.

[38] Chan DW (1992). Coping with depressed mood among Chinese medical students in Hong Kong. J Affect Disord.

[39] Sharif Nia H, Pahlevan Sharif S, Goudarzian AH, Allen KA, Jamali S, Heydari Gorji MA (2017). The Relationship between Religious Coping and Self-Care Behaviors in Iranian Medical Students. J Relig Health.

[40] Nechita D, Vasile DL, Nechita F, Strunoiu LM, Albulescu D-M (2019). Trait anxiety and coping in first year medical students. Rom J Morphol Embryol.

[41] Francis B, Gill JS, Yit Han N, Petrus CF, Azhar FL, Ahmad Sabki Z, Said MA, Ong Hui K, Chong Guan N, Sulaiman AH (2019). Religious Coping, Religiosity, Depression and Anxiety among Medical Students in a Multi-Religious Setting. Int J Environ Res Public Health.

[42] Demiral Yilmaz N, Sahin H, Nazli A (2020). International medical students' adaptation to university life in Turkey. Int J Med Educ.

[43] Mohammed A, Mostafa A, Hussien Z, Redah MH, Adnan T, Mohammed H (2020). Prevalence of Stress and Coping Behaviors among Medical Students at University of AL-Qadisiyah. Medico Legal Update.

[44] Abdulghani HM, Sattar K, Ahmad T, Akram A (2020). Association of COVID-19 Pandemic with undergraduate Medical Students' Perceived Stress and Coping. Psychol Res Behav Manag.

[45] Al-Dubai SA, Al-Naggar RA, Alshagga MA, Rampal KG (2011). Stress and coping strategies of students in a medical faculty in malaysia. Malays J Med Sci.

[46] Shaban IA, Khater WA, Akhu-Zaheya LM (2012). Undergraduate nursing students' stress sources and coping behaviours during their initial period of clinical training: a Jordanian perspective. Nurse Educ Pract.

[47] Seyedfatemi N, Tafreshi M, Hagani H (2007). Experienced stressors and coping strategies among Iranian nursing students. BMC Nurs.

[48] Tully A (2004). Stress, sources of stress and ways of coping among psychiatric nursing students. J Psychiatr Ment Health Nurs.

[49] ZupiriaGorostidi X, HuitziEgilegor X, Jose AlberdiErice M, Jose UrangaIturriotz M, EizmendiGarate I, Barandiaran Lasa M, SanzCascante X (2007). Stress sources in nursing practice Evolution during nursing training. Nurse Educ Today.

[50] Nolan G, Ryan D (2008). Experience of stress in psychiatric nursing students in Ireland. Nurs Stand.

[51] Newbury-Birch D, Walshaw D, Kamali F (2001). Drink and drugs: from medical students to doctors. Drug Alcohol Depend.

[52] Park CL, Adler NE (2003). Coping style as a predictor of health and well-being across the first year of medical school. Health Psychol.

[53] Chaabane S, Chaabna K, Bhagat S, Abraham A, Doraiswamy S, Mamtani R, Cheema S (2021). Perceived stress, stressors, and coping strategies among nursing students in the Middle East and North Africa: an overview of systematic reviews. Syst Rev.

[54] Saravanan C, Wilks R (2014). Medical students' experience of and reaction to stress the role of depression and anxiety. ScientificWorldJournal.

[55] Carson AJ, Dias S, Johnston A, McLoughlin MA, O'Connor M, Robinson BL, Sellar RS, Trewavas JJC, Wojcik W (2000). Mental Health in Medical Students a Case Control Study Using the 60 Item General Health Questionnaire. Scott Med J.

[56] Dyrbye LN, Thomas MR, Shanafelt TD (2006). Systematic Review of Depression, Anxiety, and Other Indicators of Psychological Distress Among U.S. and Canadian Medical Students. Academic Medicine.

[57] Jurkat H, Höfer S, Richter L, Cramer M, Vetter A (2011). Quality of life, stress management and health promotion in medical and dental students A comparative study. Dtsch Med Wochenschr.

[58] Selb M, Kohler F, Robinson Nicol MM, Riberto M, Stucki G, Kennedy C, Üstün B (2015). ICD-11: a comprehensive picture of health, an update on the ICD-ICF joint use initiative. J Rehabil Med.

[59] Fares J, Al Tabosh H, Saadeddin Z, El Mouhayyar C, Aridi H (2016). Stress, Burnout and Coping Strategies in Preclinical Medical Students. N Am J Med Sci.

[60] Ke X, Liu C, Li N (2010). Social support and Quality of Life: a cross-sectional study on survivors eight months after the 2008 Wenchuan earthquake. BMC Public Health.

[61] Trucchia SM, Lucchese MS, Enders JE, Fernández AR (2013). Relationship between academic performance, psychological well-being, and coping strategies in medical students. Rev Fac Cien Med Univ Nac Cordoba.

[62] Newbury-Birch D, White M, Kamali F (2000). Factors influencing alcohol and illicit drug use amongst medical students. Drug Alcohol Depend.

[63] Tyssen R, Vaglum P, Aasland OG, Grønvold NT, Ekeberg O (1998). Use of alcohol to cope with tension, and its relation to gender, years in medical school and hazardous drinking: a study of two nation-wide Norwegian samples of medical students. Addiction.

[64] Jackson ER, Shanafelt TD, Hasan O, Satele DV, Dyrbye LN (2016). Burnout and Alcohol Abuse/Dependence Among U.S. Medical Students. Acad Med.

[65] Hackney CH, Sanders GS (2003). Religiosity and Mental Health: A Meta-Analysis of Recent Studies. J Sci Study Relig.

[66] Ano GG, Vasconcelles EB (2005). Religious coping and psychological adjustment to stress: a meta-analysis. J Clin Psychol.

[67] Pargament KI, Tarakeshwar N, Ellison CG, Wulff KM (2001). Religious Coping Among the Religious: The Relationships Between Religious Coping and Well-Being in a National Sample of Presbyterian Clergy, Elders, and Members. J Sci Study Relig.

[68] Pargament KI, Koenig HG, Perez LM (2000). The many methods of religious coping: development and initial validation of the RCOPE. J Clin Psychol.

[69] Coyne JC, DeLongis A (1986). Going beyond social support: the role of social relationships in adaptation. J Consult Clin Psychol.

[70] Russell DW, Cutrona CE (1991). Social support, stress, and depressive symptoms among the elderly: test of a process model. Psychol Aging.

[71] Uchino BN, Uno D, Holt-Lunstad J (1999). Social Support, Physiological Processes, and Health. Curr Dir Psychol Sci.

[72] Ye Z, Yang X, Zeng C, Wang Y, Shen Z, Li X, Lin D (2020). Resilience, Social Support, and Coping as Mediators between COVID-19-related Stressful Experiences and Acute Stress Disorder among College Students in China. Appl Psychol Health Well Being.

[73] Zvauya R, Oyebode F, Day EJ, Thomas CP, Jones LA (2017). A comparison of stress levels, coping styles and psychological morbidity between graduate-entry and traditional undergraduate medical students during the first 2 years at a UK medical school. BMC Res Notes.

[74] Ashton CH, Kamali F (1995). Personality, lifestyles, alcohol and drug consumption in a sample of British medical students. Med Educ.

[75] Pickard M, Bates L, Dorian M, Greig H, Saint D (2000). Alcohol and drug use in second-year medical students at the University of Leeds. Med Educ.

[76] Baldwin DC, Hughes  PH, Conard SE, Storr CL, Sheehan DV (1991). Substance use among senior medical students. A survey of 23 medical schools. Jama.

[77] Yousafzai AW, Ahmer S, Syed E, Bhutto N, Iqbal S, Siddiqi MN, Zaman M (2009). Well-being of medical students and their awareness on substance misuse: a cross-sectional survey in Pakistan. Ann Gen Psychiatry.

[78] Durkin SR, Bascomb A, Turnbull D, Marley J (2003). Rural origin medical students: how do they cope with the medical school environment?. Aust J Rural Health.

[79] Soliman M (2014). Perception of stress and coping strategies by medical students at King Saud University, Riyadh, Saudi Arabia. Journal of Taibah University Medical Sciences.

[80] Stewart SM, Betson C, Lam TH, Marshall IB, Lee PW, Wong CM (1997). Predicting stress in first year medical students: a longitudinal study. Med Educ.

[81] Yilmaz ND, Sahin H, Nazli A (2020). International medical students’ adaptation to university life in Turkey. Int J Med Educ.

