# Correction to: Effective coping strategies utilised by medical students for mental health disorders during undergraduate medical education - a scoping review

**DOI:** 10.1186/s12909-022-03223-y

**Published:** 2022-03-10

**Authors:** Kamran Sattar, Muhamad Saiful Bahri Yusoff, Wan Nor Arifin, Mohd Azhar Mohd Yasin, Mohd Zarawi Mat Nor

**Affiliations:** 1 Department of Medical Education, School of Medical Sciences, Universiti Sains Malaysia, Kelantan, Malaysia; 2 Biostatistics and Research Methodology Unit, School of Medical Sciences, Universiti Sains Malaysia, Kelantan, Malaysia; 3 Department of Psychiatry, School of Medical Sciences, Universiti Sains Malaysia, Health Campus, Kelantan, Malaysia

**Correction to: BMC Med Educ 22, 121 (2022)**

**https://doi.org/10.1186/s12909-022-03185-1**

Following publication of the original article [1], we have been informed that the caption for Figure 1 and Figure 2 is wrongly placed. It should be switched.

The original article has been corrected.
