# Supplementary material for: Effective coping strategies utilised by medical students for mental health disorders during undergraduate medical education-a scoping review
Source: BMC Med Educ. 2022 Feb 23;22:121. doi: 10.1186/s12909-022-03185-1 (PMC8863569; doi:10.1186/s12909-022-03185-1)
Supplement: Supplementary file 1 — Additional file 1. [file 12909_2022_3185_MOESM1_ESM.docx]

**Supplementary file**

**Appendix 1:** Overview of the included studies

| **S.no** | **Author** | **Year** | **Location** | **Aims/objective/purpose** | **Sample/ study design/tools** | **CSs used** |
| --- | --- | --- | --- | --- | --- | --- |
| 1 | TM Wolf, JM Faucett, HM Randall and PM Balson [24] | 1988 | USA | “To gather data on the stress and coping of medical students in order to design a health promotion and wellness program”. | - 71 medical students - Retrospective - Questionnaire | - Planful problem-solving (deliberate problems-focused efforts to alter the situation) |
| 2 | DW Chan [38] | 1992 | Hong Kong | “To assess depressed mood and coping activities”. | - 95 medical students - Beck Depression Inventory (BDI) - Coping Strategies Scales (COSTS) | - Problem-focused activities - Avoiding and denying problems and containing emotions, |
| 3 | KJ Moffat, A McConnachie, S Ross and JM Morrison [32] | 2004 | UK | “To examine the prevalence of psychological morbidity, sources of stress, and coping mechanisms”. | - 275 medical students - Longitudinal cohort questionnaire survey - 12-item General Health Questionnaire (GHQ-12), 9-item list of potential stressors, Brief COPE | - Increased student feedback and guidance about progress throughout the year and the provision of adequate learning resources may reduce student stress. |
| 4 | BT Shaikh, A Kahloon, M Kazmi, H Khalid, K Nawaz, N Khan and S Khan [35] | 2004 | Pakistan | “To assess the perception of stress amongst medical students and their CSs”. | - 264 medical students - A cross-sectional study using a semi-structured self-administered questionnaire | - Spending time with friends - Sleep Music - Sports - Isolation - Study |
| 5 | CT Sreeramareddy, PR Shankar, VS Binu, C Mukhopadhyay, B Ray and RG Menezes [13] | 2007 | Nepal | “To assess the prevalence of psychological morbidity, sources, and severity of stress and CSs among medical students”. | - 407 medical students - Cross-sectional - A 24-item questionnaire, Brief COPE | - Positive reframing, - Planning, - Acceptance, - Active coping, - Self-distraction - Emotional support |
| 6 | C Shah, RS Trivedi, J Diwan, R Dixit and AK Anand [28] | 2009 | India | “To determine what the most prevalent sources of stressors among our students are, and to evaluate the coping mechanisms adopted by them”. | - 126 medical students - Questionnaire | - Talking with friends - Go to sleep - Playing and watching sports - Go to home - Calling parents with phone - Planning of study/searching for good materials - Movies/internet/video games/cultural activities/hobbies - Seek help from seniors - Seek help from teachers - Yoga - Smoking tobacco chewing/physical exercise |
| 7 | J Shapiro and D Lie [25] | 2009 | USA | “To examine medical students’ writing about a hypothesized traumatic physician-patient encounter in relation”. | - 89 - Objective Structured Clinical Examination (OSCE) that included a writing exercise | - **Active (**moving on). - **Accepting** (accepting professional limitations). - **Communicative** (talking to others). - **Self-focused** (taking time off, caring for oneself). - **Detached** (not thinking about the situation; not getting too close to patients; focusing on technical aspects). |
| 8 | S Cherkil, SJ Gardens and DK Soman [29] | 2013 | India | “To find the association between coping styles and stress in undergraduate medical students”. | - 490 (303 medical 187 dental) - A cross-sectional study design - Source and Severity of Stress Scale, Brief COPE | - Support - Non-cope - Blame - Humour - Substance |
| 9 | S Sen, D Pal, S Hazra and GK Pandey [30] | 2015 | India | “To measure the spiritual health status of the study population, describe the coping skills used by them in crisis situations, identify the sociodemographic factors associated with their spiritual health, and to determine the association of spiritual health status of the study population and their coping skills”. | - 362 medical students - Cross-sectional observational study - Questionnaire | **Adaptive coping**   - Active coping - Seeking emotional support - Seeking instrumental support - Planning - Positive reframing   **Maladaptive coping**   - Denial - Behavioural Disengagement - Substance use - Self-distraction - Self-blame   **Other common strategies**   - Acceptance - Turning to religion - Humour - Venting |
| 10 | JH Schiller, RB Stansfield, DC Belmonte, JA Purkiss, RM Reddy, JB House and SA Santen [4] | 2018 | USA | “To explore differences in students’ use of active, problem-solving strategies and emotional, inwardly directed approaches; the change in CSs used during medical school; and CS impact on performance”. | - 183 - 22 item Ways of Coping Scale (WCS) Questionnaire | **Active ways of coping**   - Seeking Social Support - Planful Problem Solving - Confrontive Coping - Positive Reappraisal   **Emotional ways of coping**   - Escape Avoidance - Distancing - Self-Control - Accepting Responsibility |
| 11 | NK Balaji, PS Murthy, DN Kumar and S Chaudhury [31] | 2019 | India | “To study the factors associated with stress, anxiety, and coping states in students”. | - 200 - Prospective, longitudinal study - Questionnaire for perceived stress scale, Hamilton Anxiety Rating Scale, Brief COPE inventory, Sources of stress questionnaire | - Active coping - Avoidant coping |
| 12 | F Rashid, Z Shahid, I Atif, S Wazir, M Khalid and F Hamid [34] | 2019 | Pakistan | “To determine the frequency of various coping strategies adopted against depression, anxiety and stress among medical students”. | - 572 medical students - Cross-sectional - Brief COPE | - Religion - Planning - Self-distraction - Positive reframing - Active coping - Acceptance - Self-blaming - Instrumental support - Emotional support - Humour - Behaviour disengagement - Venting - Denial - Substance abuse |
| 13 | V Steiner-Hofbauer and A Holzinger [3] | 2020 | Austria | “To evaluate the prevalence of stress and depression and the efficacy of CSs in undergraduate medical students”. | - 589 medical students - Cross-sectional study - Stress and coping questionnaire, Depression screening, Substance use questionnaire, Leisure time activities questionnaire | - Positive thinking - Active coping - Faith/religion - Social support - Leisure time activities - Substance use |
| 14 | G Thompson, RB McBride, CC Hosford and G Halaas [26] | 2016 | USA | “To determine the prevalence of burnout and depression among medical students at a rural medical school”. | - 161 medical students - Cross-sectional research design - Patient Health Questionnaire (PHQ) | - A majority (n D 104; 64.6%) used a combination of approach and avoidant CSs - More avoidant CSs - Balanced coping strategies - More approach CSs |
| 15 | H Sharif Nia, S Pahlevan Sharif, AH Goudarzian, KA Allen, S Jamali and MA Heydari Gorji [39] | 2017 | Iran | “To determine the relationship between religious coping methods (i.e., positive and negative religious coping) and self-care behaviours in Iranian medical students”. | - 335 medical students - Cross-sectional design - Standard questionnaire of religious coping methods - Questionnaire of self-care behaviours assessment | - Positive religious coping - Negative religious coping |
| 16 | R Erschens, T Loda, A Herrmann-Werner, KE Keifenheim, F Stuber, C Nikendei, S Zipfel and F Junne [36] | 2018 | Germany | “To explore the functional and dysfunctional CSs of medical students with regard to their respective burnout factors”. | - 597 medical students - Self-administered - Maslach Burnout Inventory-Student Version (MBI-SS) | - **Functional Coping Strategies:** - Seeking support from friends - Seeking support from family - Doing relaxing exercise - Doing sports - Seeking support from fellow students - **Non-functional CSs:** - Taking tranquillizers - Taking stimulants - Drinking alcohol - Withdrawal and ruminating - Playing games on PC or mobile phone |
| 17 | M Akhtar, BK Herwig and FA Faize [37] | 2019 | Germany | “To explore the predictive role of dispositional coping styles in determining the level of depression and anxiety among international l medical students in Germany”. | - 122 medical students - Cross-sectional study - Major Depression Inventory, Beck Anxiety Inventory, Problem-Focused Styles of Coping Inventory | - Reflective coping - Suppressive coping - Reactive coping Reflective coping |
| 18 | B Francis, JS Gill, N Yit Han, CF Petrus, FL Azhar, Z Ahmad Sabki, MA Said, K Ong Hui, N Chong Guan and AH Sulaiman [41] | 2019 | Malaysia | “To determine the prevalence of depressive and anxiety symptoms among medical students and the association between religious coping, religiosity and socio-demographic factors with anxiety and depressive symptoms”. | - 622 medical students - Cross-sectional design - Malay version of the Duke Religious Index (DUREL-M), the Malay version of the Brief Religious Coping Scale (Brief RCOPE) and the Malay version of Hospital and Anxiety Depression Scale (HADS-M) | - Negative religious coping - Positive religious coping |
| 19 | D Nechita, DL Vasile, F Nechita, LM Strunoiu and D-M Albulescu [40] | 2019 | Romania | “To highlight the types of anxiety and the CSs employed by the students at the Medicine, Pharmacy and Nursing Faculties, at the same time comparing them with the general population”. | - 333 medical students - Endler Multidimensional Anxiety Scales–Trait (EMAS-T) and Social Anxiety Scale–Trait (SAS-T) and two coping measuring instruments, Cognitive Emotion Regulation Questionnaire (CERQ) and Strategic Approach to Coping Scale (SACS) | - Maladaptive cognitive-emotional coping mechanisms; - Rumination and acceptance |
| 20 | T Trivate, AA Dennis, S Sholl and T Wilkinson [33] | 2019 | UK | “To explore students' experiences with patient death in a UK context. These include CSs, support from faculty following patient death and the relationship between these experiences and learning”. | - 12 medical students - Qualitative study - An online questionnaire with narrative responses | - Internal CSs: - Personal re-interpretation of the experiences - Normalization - Enduring the negative emotion - Staying busy - External CSs: - Speaking to peers and to near peers - Speaking to consultants - Speaking to family |
| 21 | N Demiral Yilmaz, H Sahin and A Nazli [42] | 2020 | Turkey | “To determine the adaptation process of international medical school students to university life in Turkey”. | - Students (n=127) - The mixed-method design study - Student Adaptation to College Questionnaire (SACQ) - Brief COPE. - Focus group interviews | - Acceptance - Self-blame - Use of instrumental and emotional support - Active coping - Positive reframing - Behavioural disengagement, - Self-distraction - Substance use |
| 22 | N Shoua-Desmarais, H von Harscher, M Rivera, T Felix, N Havas, P Rodriguez, G Castro and E Zwingli [27] | 2020 | USA | “To preliminarily examine these relationships (between CSs and burnout) among first-year medical students”. | - 167 medical students - Cross-sectional - COPE inventory - Maslach Burnout Inventory-Human Services SurveyMBI-HS | - Adaptive CS of planning - Aaptive CS of positive re-interpretation/growth |
| 23 | A Mohammed, A Mostafa, Z Hussien, MH Redah, T Adnan and H Mohammed [43] | 2020 | Iraq | “To evaluate the prevalence of stress and coping behaviours among medical students”. | - 203 medical students - A descriptive cross-sectional questionnaire-based study - Medical Student Stress Questionnaire (MSSQ) - Coping Behaviour Inventory (CBI) | - Problem-solving behaviours - Optimistic coping behaviours - Transference behaviours - Avoidance behaviours |
| 24 | HM Abdulghani, K Sattar, T Ahmad and A Akram [44] | 2020 | Saudi Arabia | “To determine the effect of the current pandemic on undergraduate medical students’ learning. We also explored the association of their stress level with CSs, educational, and psychological variables”. | - 243 medical students - Cross-sectional design study - A self-administered questionnaire - Kessler 10- Psychological distress questionnaire | - Regular exercise - Watching online movies - Playing online games - Online fun with family and friends - Religious activities - Learning to live in the current COVID situation and accept it - Refusing to believe the current COIVD situation. - Online help advise friend and experts to feel good. |
