# Supplementary material for: Effective coping strategies utilised by medical students for mental health disorders during undergraduate medical education-a scoping review
Source: BMC Med Educ. 2022 Feb 23;22:121. doi: 10.1186/s12909-022-03185-1 (PMC8863569; doi:10.1186/s12909-022-03185-1)
Supplement: Supplementary file 2 — Additional file 2. [file 12909_2022_3185_MOESM2_ESM.docx]

**Supplementary file**

**Appendix 2**

**SEARCH STRATEGY**

| **PubMed** | MeSH terms utilized [Coping]= Psychological Adaptation, Adjustment, Coping Skills, [Mental Wellbeing] = Well Being, Adolescent, Well-being, Adolescent, and [Medical].  Year 1986-2021 |
| --- | --- |
| **Scopus** | TITLE ( coping  AND skills )  AND  ( LIMIT-TO ( DOCTYPE ,  "ar" ) )  AND  ( LIMIT-TO ( SUBJAREA ,  "MEDI" )  OR  LIMIT-TO ( SUBJAREA ,  "PSYC" )  OR  LIMIT-TO ( SUBJAREA ,  "HEAL" ) )  AND  ( LIMIT-TO ( LANGUAGE ,  "English" ) )  AND  ( LIMIT-TO ( SRCTYPE ,  "j" ) )  AND  ( LIMIT-TO ( EXACTKEYWORD ,  "Human" ) ) |
| **Google Scholar** | (with **all** of the words)= allintitle: Coping Strategy -1986-2021 |
